# InverseMuscleNET: Alternative Machine Learning Solution to Static Optimization and Inverse Muscle Modeling

**DOI:** 10.3389/fncom.2021.759489

**Published:** 2021-12-23

**Authors:** Ali Nasr, Keaton A. Inkol, Sydney Bell, John McPhee

**Affiliations:** Department of Systems Design Engineering, University of Waterloo, Waterloo, ON, Canada

**Keywords:** recurrent neural network (RNN), muscle modeling, electromyography, static optimization, inverse problems solution

## Abstract

InverseMuscleNET, a machine learning model, is proposed as an alternative to static optimization for resolving the redundancy issue in inverse muscle models. A recurrent neural network (RNN) was optimally configured, trained, and tested to estimate the pattern of muscle activation signals. Five biomechanical variables (joint angle, joint velocity, joint acceleration, joint torque, and activation torque) were used as inputs to the RNN. A set of surface electromyography (EMG) signals, experimentally measured around the shoulder joint for flexion/extension, were used to train and validate the RNN model. The obtained machine learning model yields a normalized regression in the range of 88–91% between experimental data and estimated muscle activation. A sequential backward selection algorithm was used as a sensitivity analysis to discover the less dominant inputs. The order of most essential signals to least dominant ones was as follows: joint angle, activation torque, joint torque, joint velocity, and joint acceleration. The RNN model required 0.06 s of the previous biomechanical input signals and 0.01 s of the predicted feedback EMG signals, demonstrating the dynamic temporal relationships of the muscle activation profiles. The proposed approach permits a fast and direct estimation ability instead of iterative solutions for the inverse muscle model. It raises the possibility of integrating such a model in a real-time device for functional rehabilitation and sports evaluation devices with real-time estimation and tracking. This method provides clinicians with a means of estimating EMG activity without an invasive electrode setup.

## 1. Introduction

**Figure 1:**
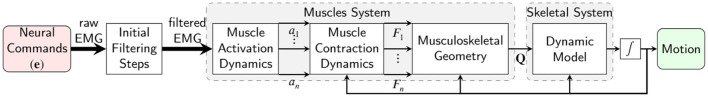
Forward simulation of a musculoskeletal system with multiple muscles. **α** and **F** are the activation signal and the muscle force in the muscle dynamic model, respectively.

Muscle contractions generate tension and, as a result, a moment about the human joint. Knowing the muscle forces or activation signals during human movement can assist in understanding the underlying biomechanical systems (Crowninshield and Brand, 1981). These analyses can improve movement performance, especially for athletes and patients (Laschowski et al., 2018). Forward dynamic simulations of a given musculoskeletal model, driven via muscle activations, yield calculated motions (Figure 1) (Ezati et al., 2019). As depicted in Figure 1, first, the raw EMG goes through initial filtering steps and a muscle activation dynamic model (Manal and Buchanan, 2003; Desplenter and Trejos, 2018). Second, the resultant activation converts to muscle forces using muscle contraction dynamics (Heitmann et al., 2012; Heidlauf and Rohrle, 2014; Desplenter and Trejos, 2018). Third, the muscle forces convert to joint torque using the musculoskeletal geometry model (Hammer et al., 2019). Finally, the joint torque is used in the forward dynamic simulation of the torque-driven skeletal model to obtain the motion (Mehrabi and McPhee, 2019).

**Figure 2:**
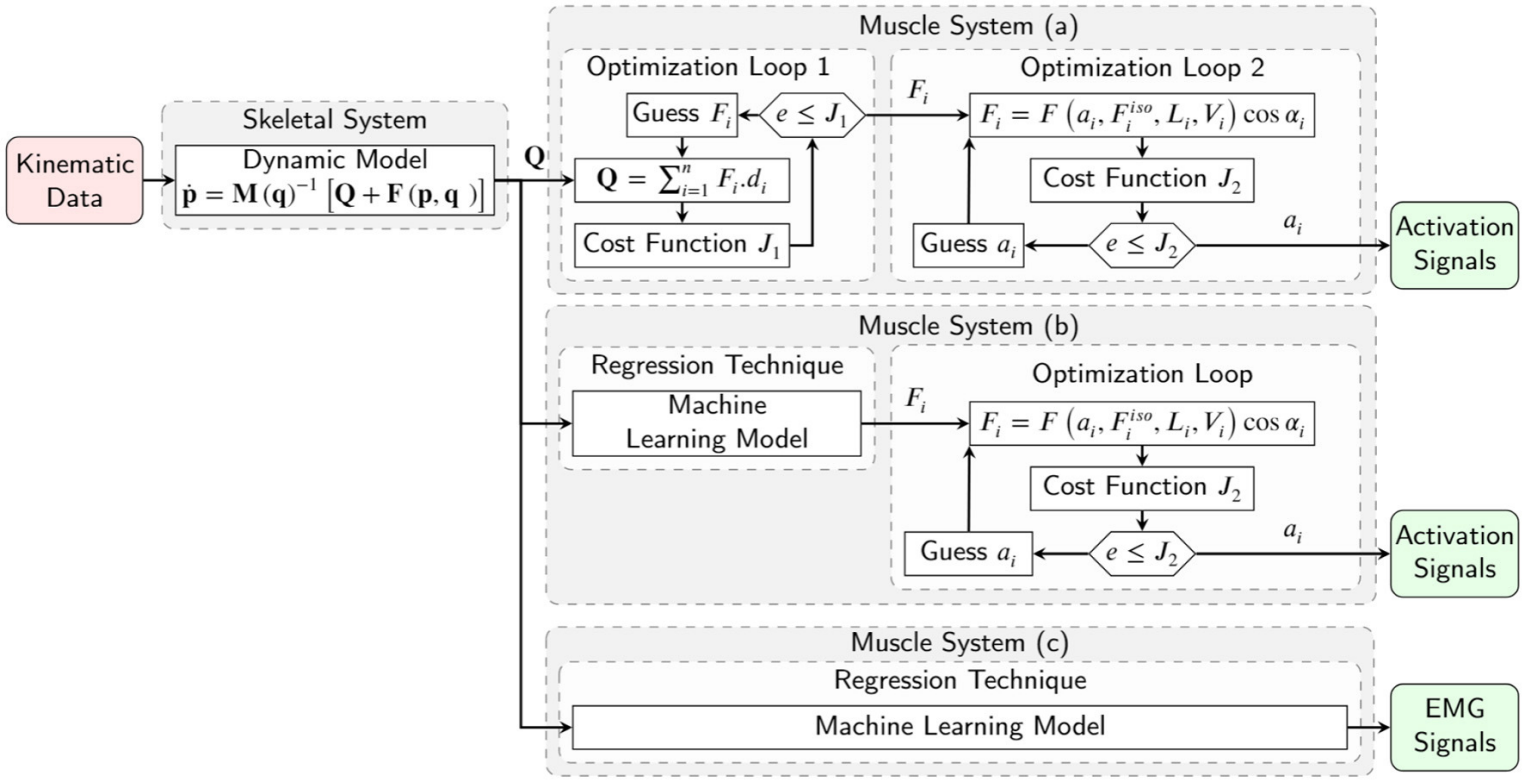
Inverse simulation of a musculoskeletal system with multiple muscles, using **(a)** conventional two optimization loops for estimation of muscle forces and activation signals, **(b)** machine learning model for muscle force estimation and one optimization loop for activation signals, and **(c)** machine learning model for activation signals estimation.

Conversely, the inverse dynamic problem yields either (I) muscle tensions or (II) EMG signals from a predefined kinematic motion, introducing a redundancy resolution problem (Anderson and Pandy, 2001; Miller et al., 2013; Shourijeh and McPhee, 2014; Shourijeh et al., 2017; Bailly et al., 2021). This redundancy is due to the number of participating muscles being greater than the number of joint degrees of freedom (Hirashima and Oya, 2016). The redundancy resolution problem is commonly formalized as an optimization approach to find multiple muscle tension combinations for a given joint torque (Anderson and Pandy, 2001; Shourijeh and McPhee, 2014; Bailly et al., 2021). This optimization minimizes a function of muscle forces, muscle activations, and/or metabolic energy expenditure (Erdemir et al., 2007; Shourijeh and McPhee, 2014). Various static and dynamic optimization formulations have been proposed by Anderson and Pandy (2001), Heintz and Gutierrez-Farewik (2007), Shourijeh et al. (2017), Ezati et al. (2019), and Shourijeh and McPhee (2014) (Figure 2a). Generally, for model simplification of the optimization problem, four assumptions have been considered (Winter, 2009): (A) there is no predefined history of the co-contraction pattern for the modeled muscles (assuming previous values have no impact), (B) the maximum muscle forces are unaltered by the muscle geometry, (C) muscles do not have dynamics and may produce any tension immediately, and (D) the musculoskeletal system satisfies a cost (Anderson and Pandy, 2001; Erdemir et al., 2007; Shourijeh and McPhee, 2014) (which is a function of muscle stresses, muscle activations, or metabolic energy expenditure). However, the mentioned assumptions do not support the observed nature of muscles (Winters, 1990; Arjmand et al., 2013; Cecchini et al., 2014; Vilimek, 2014; Dao, 2019) as muscles follow a co-contraction pattern (Michaud et al., 2020), are defined by a dynamic model (Siebert et al., 2008), and have a geometry that impacts the maximum muscle forces (Heidlauf and Rohrle, 2014; Desplenter and Trejos, 2018).

In conventional dynamic approaches, muscle activations or EMG signals are determined by an optimization algorithm (Figure 2a) (Anderson and Pandy, 2001; Shourijeh and McPhee, 2014; Shourijeh et al., 2017). Concurrently, the muscle tensions and joint moments are calculated by an EMG-driven model simulation. The optimization cost function is computed using a comparison of the joint moments with desired joint moments, calculated by the skeletal model's inverse dynamic simulation (Michaud et al., 2020). No inverse muscle model is used, and optimization tries to find the inputs of the forward dynamic muscle model to achieve specific joint moments (Moissenet et al., 2019). The issues here lie in our approximation of complex skeletal muscle mechanics, which often requires modeling assumptions and sophisticated geometry/wrapping models (Winter, 2009; Ellis et al., 2018; Hammer et al., 2019). Sensitivities and difficulties in fitting and identifying the relevant muscle parameters (Millard et al., 2019; Serrancolí et al., 2020), in addition to uncertainties related to experimental data, add further challenges to this process (Valente et al., 2014). Due to the mentioned assumptions and challenges, the simulation results in estimated muscle activations that may not be correct (Norman-Gerum and McPhee, 2018).

Machine learning is an instrument or model for solving complicated mathematical problems without knowing the analytical relationship between the inputs and outputs (Rane et al., 2019). Supervised machine learning offers an alternative solution to the optimization, leading to the easing of muscle redundancy, complexity, and pattern estimation of muscle tensions (or EMG signals) within a musculoskeletal model (Rane et al., 2019; Sohane and Agarwal, 2020). This methodology substitutes the mathematical equations in musculoskeletal modeling with a network of interconnected artificial neurons imitating central nervous system function. Experimental data is used to train the model to find the mapping from joint moment time-series to a pattern of muscle tensions or EMG signals. This approach offers a computationally efficient method; however, it requires collecting and processing a significant amount of experimental data as well as optimizing the machine learning model configuration for the highest prediction accuracy, mapping efficiency, and real-time estimation ability.

Recently, machine learning models have been developed to estimate skeletal muscle tensions without explicit modeling of the physical muscle behaviors (Arjmand et al., 2013; Cecchini et al., 2014; Vilimek, 2014; Dao, 2019; Rane et al., 2019). Arjmand et al. (2013) constructed artificial neural networks (ANNs) to predict the trunk muscle forces required during static lifting. They used five input variables: thorax flexion angle, load magnitude, the anterior and lateral distances of the reaching task, and the load handling technique. They disseminated that ANNs are more accurate than a regression-based mapping of input-output relationships. Cecchini et al. (2014) also used ANNs to predict muscle force patterns for an athlete cycling. They validated the ANN against an independent validation set and compared two alternative approaches using the Bland-Altman method (Cecchini et al., 2014). Vilimek (2014) used an ANN model for estimating the musculotendon forces around the elbow joint during a flexion/extension movement. Here, the network model's input parameters were the morphological and anatomical musculotendon parameters as well as the measured activation level. The author used that ANN for one specific muscle, which showed better results than the ANN for general muscles (Vilimek, 2014). Instead of the previous ANN models (Arjmand et al., 2013; Cecchini et al., 2014; Vilimek, 2014) for considering the dynamic temporal relationships of the muscle forces, which is a time-series forecasting problem, recurrent deep neural networks (RDNNs) have been used by Dao (2019). Dao (2019) used a long short-term memory model as an RDNN to approximate muscle forces from kinematic data. Rane et al. (2019) trained a convolutional neural network on a set of kinematic, kinetic, and electromyographic measurements from 156 subjects to predict the muscle internal force magnitudes.

Many previous studies (Arjmand et al., 2013; Cecchini et al., 2014; Vilimek, 2014; Dao, 2019; Rane et al., 2019) have predicted muscle tensions only and not activations in their solutions to the muscle redundancy problem. However, the inverse muscle model was not evaluated using the machine learning model in these previous studies (Figure 2b). Commonly, using the previous solution of the estimated muscle tensions, a second optimization problem should be used to estimate the muscle activations since there is no inverse dynamic model of the muscles. Providing an inverse dynamic model of the muscles with inputs of joint moments and outputs of activation variables can offer the ability to compare the subject's EMG signals via estimated activation signals. This inverse model completes the inverse dynamic simulation of the skeletal model for biomechanical analysis, post-rehabilitation analysis, and sports engineering/optimization.

A successful study by Gonzalez-Vargas et al. (2015) developed a predictive model to generate muscle-specific excitation patterns for a given locomotion condition (i.e., speed and elevation) and a set of weightings characterizing the condition. However, the proposed pattern regression was used for cyclic gait and required subject-specific muscle weightings. To date, a couple of studies predict muscle activations using ANN machine learning and different kinds of kinematic or kinetic input signals (Heller et al., 1993; Jonic and Popovic, 1997; Prentice et al., 2001; Tibold and Fuglevand, 2015). Since the ANN's performance deteriorates with small variations in data, Sekiya et al. (2019) used the linear logistic regression model, but its generalization performance is limited. Recently, Nasr et al. (2021a) demonstrated that Recurrent Neural networks (RNN)s could outperform ANNs (forward networks) for direct muscle modeling (EMG to biomechanical signal). We plan to use an RNN to predict muscle activities from biomechanical signals (inverse to the forward model in Nasr et al., 2021a). To the best of our knowledge, the inverse muscle model using the recurrent machine learning method is novel and proposed for the first time (Figure 2c). The contributions of this paper are as follows:

1. the development of an optimized recurrent neural network configuration to estimate EMG signals from kinematic and dynamic data;
2. the evaluation and acquiring of necessary biomechanical data for the estimation, including joint angle, velocity, acceleration, joint torque, and activation torque;
3. the assessment of estimation possibility of EMG channels for a given joint;
4. the evaluation of the general and subject-based model in the estimation model; and
5. an explanation of how InverseMuscleNET can be used in applications for biomechanical analysis (inverse dynamic musculoskeletal simulation), post-rehabilitation analysis (comparison of subjects' EMG signals against those of a healthy individual via estimated activation signals), sports engineering/optimization, functional electrical stimulation control (application of the estimated healthy limb muscle activation to the functional electrical stimulation probe), and assessment and biofeedback (Gonzalez-Vargas et al., 2015).

In this paper, firstly, data preparation is introduced. Secondly, the machine learning model, configuration optimization, and evaluation algorithms are described. Finally, results are discussed and evaluated.

## 2. Data Preparation

**Figure 3:**
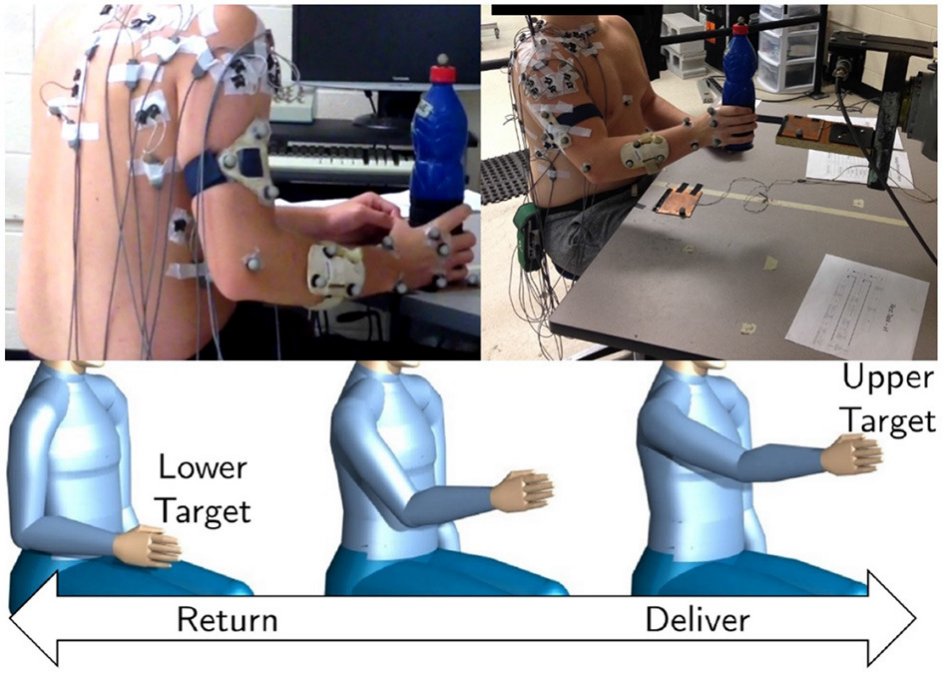
A depiction of EMG electrodes and the retroreflective position marker placements by Whittaker et al. (2018) (top). A drawing of the desired upper limb motion (bottom). The participants were required to lift and lower a weighted object between two target locations in the sagittal plane.

Experimental data of object manipulation in the sagittal plane was used for training the inverse muscle model. The protocol for data collection and preparation is discussed in the current section. The motor task is visualized in Figure 3. The experimental procedure was in accordance with the Declaration of Helsinki. The university office of research ethics approved the data collection research (ORE #: 21246).

Whittaker et al. (2018) and Whittaker et al. (2019) collected the data for a repetitive manual upper-limb task from healthy right-handed young individuals. The subjects gently lifted an object (bottle) from a lower target, placed it on the upper target, and vice versa (Figure 3). In this data collection, 17 right-handed young individuals [8 Males and 9 Females; 23±4 years; 72.25±29.85 (kg) mass; 1.66±0.16 (m) height] free of upper limb injury performed the experimental tasks.

The 3D upper-limb motion and muscle EMG signals were recorded at the same time. As suggested by Avers and Brown (2018), Noraxon Bipolar Surface circular Ag-AgCl electrodes (Noraxon Inc, Arizona, USA) with a 20 mm inter-electrode distance were used to measure the surface EMG signals from 11 locations over the following right upper-limb muscles: Serratus Anterior (SERR), Middle Deltoid (MDEL), Supraspinatus (SUPR), Infraspinatus (INFR), Posterior Deltoid (PDEL), Pectoralis Major (PECC), Latissimus Dorsi (LATS), Anterior Deltoid (ADEL), Middle Trapezius (MTRA), Upper Trapezius (UTRA), and Lower Trapezius (LTRA). The ground electrode was placed over the clavicle. A Noraxon T2000 telemetered system, TeleMyo, (Noraxon Inc, Arizona, USA), collected signals from 11 bipolar electrodes. Whittaker et al. (2018) and Whittaker et al. (2019) positioned the single bipolar electrode over each muscle. Then, signal quality was tested by examining the signal amplitude when participants stimulated the muscle through isometric contractions. The raw EMG signals were amplified, sampled at a 1,500 Hz rate, and finally, digitalized. Eight Vicon MX20+ cameras (Vicon Motion Systems, Oxford, U.K.) were used to record the 3D position of retroreflective markers attached as rigid clusters to the subjects' forearm, upper arm, and scapula segments at 50 Hz.

**Figure 4:**
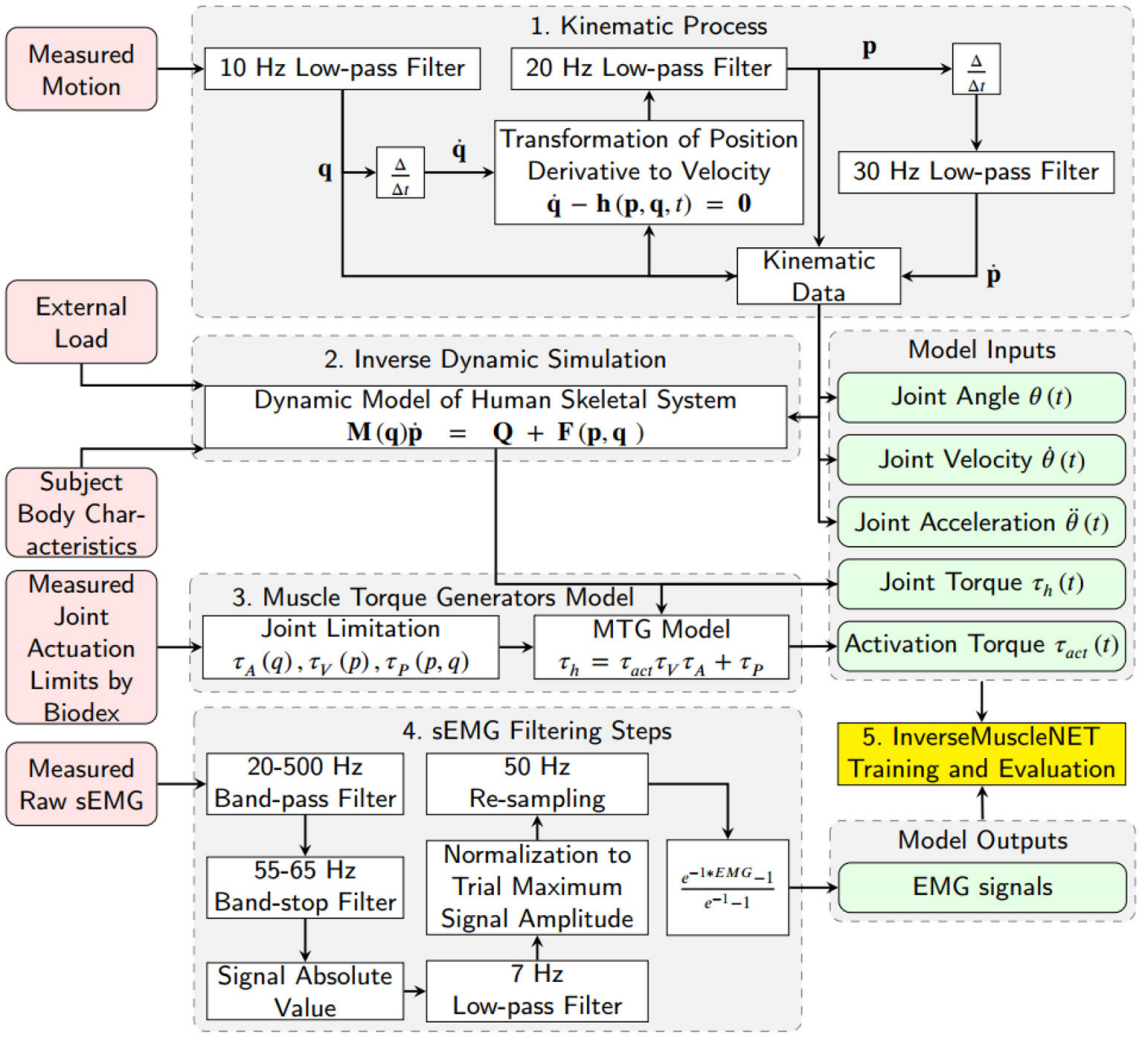
Schematic of data preparation for training InverseMuscleNET. The inputs of InverseMuscleNET are joint angle, joint velocity, joint acceleration, joint torque, and activation torque. The outputs of the InverseMuscleNET are the predicted EMG signals.

The following sections describe how the upper-limb position, external load, and EMG signals are used in the data preparation (Figure 4). These steps prepare the RNN inputs (joint angles, velocity, acceleration, joint torque, and activation torque) and the RNN outputs (filtered EMG signals).

### 2.1. Kinematic Data Estimation

A 10 Hz low-pass filter was used to eliminate the recording noise in the 3D joint angle (Euler angles for shoulder joint) data. Joint velocities were calculated using (A) the numerical first derivative of position (Euler angle), (B) the transformation of position (Euler angle) derivatives to angular velocity by Equation (1), and (C) a low-pass filter with a 20 Hz cut-off frequency. Joint accelerations were estimated using another numerical derivative (first) of the joint velocities and a 30 Hz low-pass filter. In this paper, the joint angle θ(*t*), joint velocity $\overset{∙}{\theta }\left(t\right)$, and joint acceleration $\ddot{\theta }\left(t\right)$ were defined for shoulder joint elevation and were used as three of the five biomechanical input signals for the inverse muscle mapping. In Equation (1), which was previously extracted and verified by Nasr et al. (2021b), $\overset{∙}{\text{q}}$, **h**, **p**, and **q** are the vector of all joint position derivatives, the right-hand side of the transformation function, the vector of all joint velocities and the vector of all joint angles (containing Euler angles), respectively. The number of degrees of freedom is n.


(1) $$\begin{array}{l} {\overset{∙}{\text{q}}}_{n\times 1}={\text{h}\left({\text{p}}_{n\times 1},{\text{q}}_{n\times 1},\text{t}\right)}_{n\times 1} \end{array}$$

### 2.2. Inverse Dynamics Simulation

An upper limb skeletal dynamic model was used within inverse dynamic simulations to estimate the required joint moments. The model had 3-DoF of rotation at the shoulder, only flexion/extension at the elbow, axial rotation (pronation/supination) of the forearm, and no wrist joint. The upper-arm and lower arm body segment inertial parameters (BSIP) were estimated from Dumas et al. (2007) and adjusted with subject mass and height. The mentioned dynamic model in Equation (2) was previously extracted and verified by Nasr et al. (2021b). In this paper, the shoulder elevation torque was defined as the human joint torque *τ*_*h*_ and was used as one of the inputs of the inverse muscle mapping. In Equation (2), **M**, **F**, **Q**, and **p** are the mass matrix, the right-hand side of the dynamic equations (consisting of Coriolis, centrifugal, and gravitational effects), the applied wrench (force/torque) from muscles to the joints, and the vector of all joint accelerations, respectively.


(2) $$\begin{array}{l} {\text{M}}_{n\times n}{\overset{∙}{\text{p}}}_{n\times 1}={\text{F}}_{n\times 1}+{\text{Q}}_{n\times 1} \end{array}$$

Equation (2) is solved for Q, one component of which is *τ*_*h*_.

### 2.3. Shoulder Muscle Torque Generators Model

Nasr et al. (2021c) studied the optimum steps to filter sEMG signals when applied to machine learning of muscle models (same database) and showed that the lowest mean squared normalized error of machine learning estimation was achieved with three steps out of 1,504 possible unique methods. The steps include applying a 70 Hz high-pass filter, rectifying, and applying a 10 Hz low-pass filter. We considered the order and configuration of the mentioned steps and tuned the frequencies of filters to optimize the mapping performance.

The shoulder elevation joint torque (*τ*_*h*_) was converted to an activation torque using a Muscle Torque Generator (MTG) model (Inkol et al., 2020). The MTG models imitate components of muscle modeling, for example, the constraint of orientation and velocity of the muscle, while ignoring the possibility of actuator redundancy (McNally and McPhee, 2018; Inkol et al., 2020; Nasr et al., 2020). The MTG model used herein is represented in Equation (3) where, *τ*_*act*_, *τ*_*a*_, *τ*_*v*_, and *τ*_*p*_ are the activation torque, the function of position-scaling, the velocity-scaling, and the passive torque respectively. The position-scaling, the velocity-scaling, and the passive functions were adopted from experimental study by McNally and McPhee (2018). The activation torque *τ*_*act*_ was defined for the shoulder flexor/extensor and was used as one of the five biomechanical input signals for the inverse muscle mapping.


(3) $$\begin{array}{l} \tau_{h}=\tau_{act}\tau_{a}\tau_{v}+\tau_{p} \end{array}$$

### 2.4. EMG Data Filtering

The raw EMG data was filtered using seven steps: (1) a 20–500 Hz band-pass filter (signals lower than 20 Hz were ignored to eliminate motion artifacts, and higher than 500 Hz were ignored as they had minor power spectral density Reaz et al., 2006), (2) a 55–65 Hz band-stop filter (to reduce the 60 Hz noise from the measurement system), (3) a rectification function of the signal amplitude (with the absolute value function), (4) a 7 Hz low-pass filter (to smooth the filtered EMG signal as assessed by Nasr et al. (2021c), which was tuned from 10 to 7 Hz to optimize the mapping performance), (5) a normalization of the signal amplitude by the maximum of the trial, (6) a re-sampling function (with the 1-D data cubic interpolation method) to a 50 Hz rate, and (7) a curving function or an exponential function (to mimic the muscle activation signals in biomechanical muscle models Winters, 1990) (Figure 4).

## 3. Method

### 3.1. RNN (Recurrent Neural Network)

Machine learning is an adaptive system that trains by using interconnected nodes or neurons in a layered composition. RNNs can outperform forward networks for direct muscle modeling (EMG to biomechanical signals) (Nasr et al., 2021a). This machine learning configuration can learn the dynamic temporal relationships of a model due to its built-in recurrent signal (the feedback from the output) (Gurchiek et al., 2019). Essentially, an RNN is a deep machine learning network structure that uses information history (of inputs and outputs) to improve the network performance and estimate current outputs.

**Figure 5:**
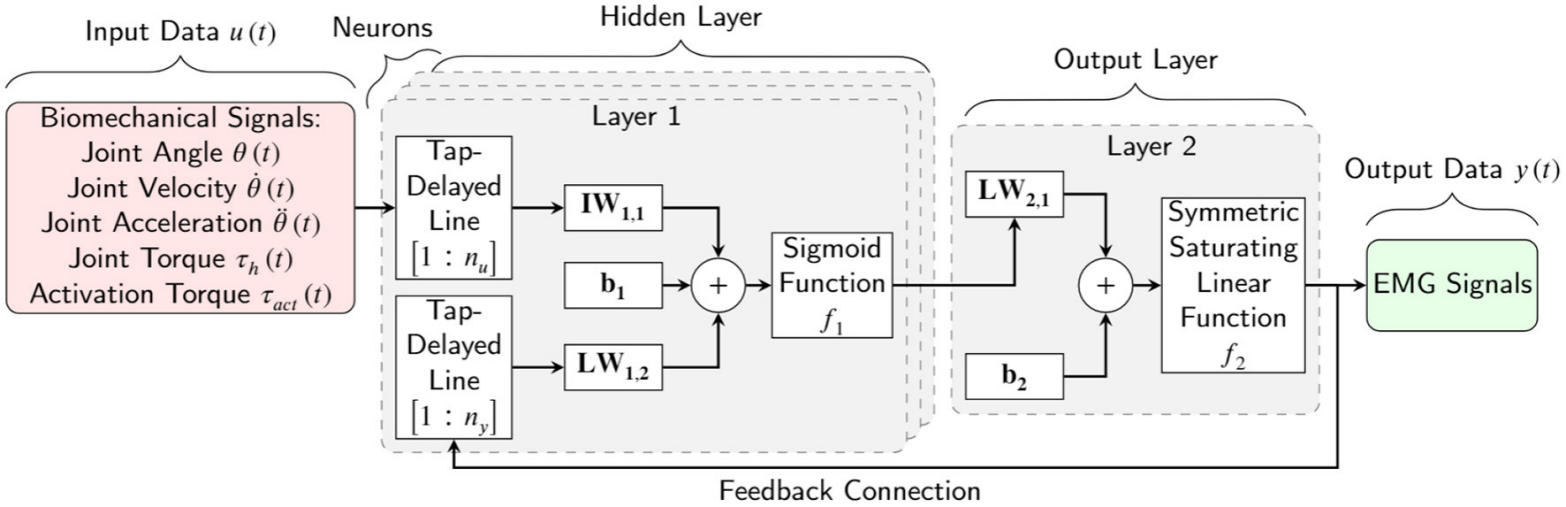
Schematic of a two-layer NARX network as an RNN learning model for EMG signal prediction. *n*_*u*_ and *n*_*y*_ are the input signal former values and the output signal former values, respectively. **IW**, **LW**, and **b** are the input weight, layer weight, and bias of one neuron, respectively.

The nonlinear autoregressive with external input neural (NARX) network was selected as an RNN learning model. NARX is a recurrent network with feedback connections that are commonly used in time-series modeling. It predicts the next output signal value of a nonlinear dynamic system. A schematic of the NARX network is shown in Figure 5 within a two-hidden-layer feedforward network. This implementation allows for multidimensional inputs and outputs. The NARX model primary equation is:


(4) $$\begin{array}{l} y\left(t\right)=f[y\left(t-1\right),\;y\left(t-2\right),\cdots \;,y\left(t-n_{y}\right),\;u\left(t\right),\;\\ \;u\left(t-1\right),\;u\left(t-2\right),\cdots \;,u\left(t-n_{u}\right)] \end{array}$$

where *y*(*t*) is the current output signal which is regressed on former values of the output signal *y*(*t*−*n*_*y*_) and former values of the input signal *u*(*t*−*n*_*u*_). *n*_*y*_ and *n*_*u*_ are the number of the previous values of the output signal and input signal. As shown in Figure 5, the feedback structure permits the network to store past information in the hidden state *y*(*t*−*n*_*y*_) and operate on current sequences *y*(*t*). Every single neuron has weights (*W*) that act as signal strength modifiers for that neuron. Every layer has a bias (*b*) that permits a shift in the activation function by adding a constant to the input. The weights and the bias are tuned throughout the learning process. The activation function in the network defines how the weighted sum of the inputs is converted into an output from the nodes in a network layer. We tested different activation functions and picked a sigmoid function for the hidden layer to provide the highest regression accuracy. Since the EMG signals had been normalized during signal processing, we proposed to use a symmetric saturating linear transfer function for the output activation function. Accordingly, the estimated output signals are always between 0 and 1.

The four primary configuration parameters of the RNN model are the: (1) number of hidden layers, (2) number of nodes in each hidden layer, (3) number of input signal former values, and (4) number of output signal former values. Using many of these parameters would lead to a high variable model that would require a time-consuming training process on a large dataset. One of the goals of this paper is to find the optimum configuration that can map kinematic and dynamic signals to EMG signals with limited training time and a limited dataset.

### 3.2. Model Inputs and Outputs

The datasets consisted of the five input signals and one output channel for 17 subjects. The inputs of the RNN model are biomechanical signals such as (1) joint angle, (2) joint velocity, (3) joint acceleration, (4) joint torque, and (5) activation torque. The first three kinematic signals (joint angle, velocity, and acceleration) are calculated in section 2.1. The last two dynamic signals (joint torque and activation torque) are calculated in sections 2.2 and 2.3, respectively. The outputs of the RNN model are the predicted EMG signals. The filtered EMG prepared in section 2.4 is used for the model output.

### 3.3. Model Training and Evaluation

There are two methods for assessing and evaluating a machine learning model: (I) subject-based estimation and (II) general-model performance. The subject-based estimation test is used as a full estimation model. It employs a data set from one subject that has not been utilized for training purposes. Thus, 14 subjects' data sets were used for training, two different subjects' data sets were used to validate the model, and one subject's data set was used in the estimation test. This method is used to optimize the RNN configuration in sections 4.1, 4.4 and is used in **Figures 9**, **12**. The general-model performance was used to evaluate the general estimation trend, general configuration, and signals' impact. The general-model utilized all subjects' data sets for training and validation purposes. This method is used for estimation analysis in section 4.2 and sensitivity analysis of input in section 4.3.

Primarily, out-of-sample (OOS) evaluation was used for the specific selection of training and validation sets. Specifically, cross-validation (CV) is a method that comparatively divides the databank into two sets of training and validation. K-fold CV acquires *k* subdivisions having an equal amount of data from the databank. Each subdivision is used as a validation set and all other subdivisions as a training set for a regression process. So, the consequent regression accuracy performance is determined by the average *k* regression accuracy (Bollen and Gu, 2005). For 17 subjects' data sets, *k* was set to 17, and the training-validation process was repeated 17 times. The average, maximum, and minimum regression accuracy for each of the 17 training-validation processes were reported in **Figure 8**. The K-fold CV was used to assess EMG signals estimation in sections 3.5 and 4.2.

To solve the nonlinear least-squares problem of the mapping model, the Levenberg-Marquardt backpropagation algorithm was used. The backpropagation algorithm calculates the Jacobian of the training performance concerning the weight and bias variables of the network. The initial adaptive mu, mu decrease factor, mu increase factor, and maximum mu value were 0.001, 0.1, 10, and 1e10, respectively. The training performance was evaluated using the mean squared normalized error (MSE) function. Parallel processing with the MEX 4 workers was used to make the training process faster. The training time was limited to 1-h, and the epoch (the number of passes of the entire training dataset to the model) was set to infinity. The linear regression of targets relative to outputs (R) was used as the mapping performance in this paper. A PC with an Intel^®^ Core™ i7-3370 CPU @ 3.40GHz and 16.0 GB of memory was used for training the RNN model.

### 3.4. Preliminary RNN Configuration Optimization

The estimation performance relies on the NARX network configuration, consisting of the number of hidden layers *n*_*l*_, the nodes in each layer *n*_*n*_, the input signal former values *n*_*u*_, and the output signal former values *n*_*y*_. These configuration parameters depend on the nature of the input signals (biomechanical signals) and the output signals (EMG signals). This research tested different possible configurations and evaluated the estimation accuracy.

For preliminary optimization of the machine learning model's configuration, all available biomechanical signals (joint angle, velocity, acceleration, torque, and activation torque) were used as the input signals. All the EMG signals were used as the output signals. The input signal sensitivity and the estimation possibility of the output signals were analyzed after the preliminary optimization of the RNN configuration.

The training condition was limited to a maximum of 1-h and 200 maximum epochs. Different NARX network configurations were tested to evaluate the mapping or pattern estimation of the RNN model. The minimum and maximum optimization limits, along with steps, are presented in Table 1.

**Table 1:** The optimization variables, limits, and steps of RNN configuration.

**Variable**	**Symbol**	**Minimum**	**Maximum**	**Step**
Number of input signal former values	*n_u_*	1	10	1
Number of output signal former values	*n_y_*	1	10	1
Number of hidden layers	*n_l_*	1	3	1
Number of nodes in each hidden layer	*n_n_*	10	50	5

### 3.5. EMG Signals Estimation

EMG signals were measured from 11 sites over the right arm muscles. The goal of this research was to estimate EMG signals from biomechanical signals. For the mentioned pick and place motion in the sagittal plane, some of the muscles may not be required for the mentioned motion. For evaluating the EMG signals estimation possibility, each EMG signal was selected as the output of the RNN model, and the estimation error was calculated as an assessment criterion. This assessment has been done using the general-model for comprehensive results and conclusions.

### 3.6. Input Biomechanical Signal Sensitivity Analysis

The joint angle, velocity, torque, and activation signal are the variables in the mathematical muscle model (Winters, 1990). To evaluate the sensitivity of EMG signals to input signals, the mapping model was an artificial neural network (ANN) with a similar configuration to the RNN (same number of hidden layers and neurons in each hidden layer). The ANN was used instead of the RNN since the evaluation of direct relationship is required for the sensitivity analysis. The direct relation means that the model is trained to map the input to the output without considering the input and output signal history. Using an RNN for sensitivity analysis has the same order of results but with higher regression accuracy. We have provided the results of sensitivity analysis with the RNN model for dominant signals in each phase.

**Figure 6:**
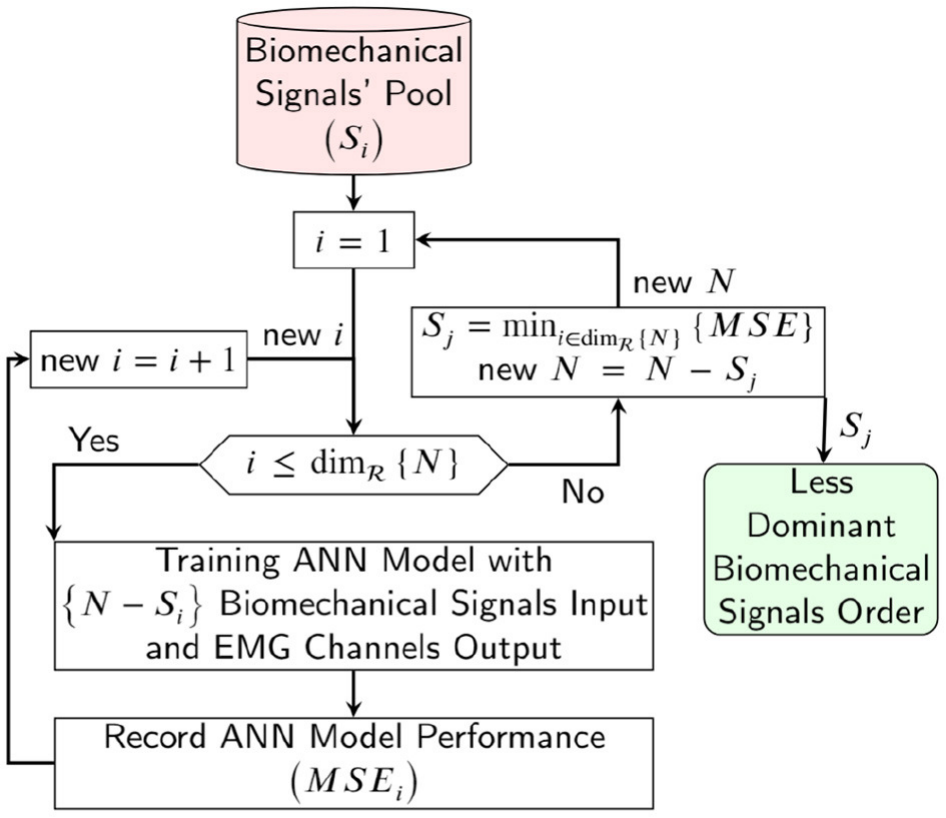
Schematic of the sequential backward selection algorithm for evaluating the sensitivity analysis and dominant biomechanical signals for estimating the EMG channels.

The sensitivity analysis to input biomechanical signals was done using a sequential backward selection algorithm for evaluating the sensitivity and dominant signals. The sensitivity analysis algorithm is shown in Figure 6 and consists of the subsequent steps:

1. Ignore the signal index *i* ∈ {*N*}, then use {*N*−*i*} of biomechanical signals as the input of the ANN model and record the mapping performance with index *i*.
2. If $i<{dim}_{R}\left\{N\right\}$ is satisfied, new i=i+1 and then step (1) is repeated.
3. The signal index j with the lowest mapping error or highest mapping performance is labeled as the least dominant biomechanical signal and is deleted from the biomechanical input pool (new {*N*} = {*N*−*j*}).
4. If the biomechanical pool is not empty, go to step (1).

### 3.7. RNN Configuration Optimization

The number of input signals was reduced using the information from section 3.6. The number of output signals was decreased by employing the results of section 3.5. Since the input and the output signals were optimized to the dominant and non-negligible ones, the initial configuration was optimized according to the new conditions of the input and output signals. The final RNN configuration optimization was done using the best results of sections 3.4 and 4.4.

## 4. Results

The optimum configuration of the RNN model, the analysis of dominant input signals, and the estimation possibility of the EMG signals are presented in the following sections.

### 4.1. Preliminary RNN Configuration

The mentioned algorithm tested 1,701 possible configurations, and the top 9 configurations are presented in Table 2. The best performance occurred when the number of layers (*n*_*l*_) was 1, not 2 or 3. Thus, the mapping of biomechanical signals to EMG signals should be limited to a linear function. Secondly, the optimal number of nodes in each layer (*n*_*n*_) was approximately 20 to 30. Fewer or extra nodes in each layer lead to lower performance. Fewer nodes means a fewer number of weights and biases, leading to lower regression accuracy. In contrast, extra nodes in a model require more time and data to train (Lee et al., 2018). Thirdly, the number of input signal former values (*n*_*u*_) was between 3 and 6 which shows that the history of biomechanical signals is important for pattern regression of EMG signals. Finally, the number of the output signal former values (*n*_*y*_) was required to be between 4 and 7 to give the best performance for the mapping. The number of output signal former values tries to model the dynamics of the EMG signals. It shows that the current EMG signal value is related to the previous EMG signal values. We have used the first RNN configuration in Table 2 for the following evaluation processes.

**Table 2:** Top performance of RNN model configurations.

**Configuration**						**Average regression (%)**			
***n_l_***	***n_n_***	***n_u_***	***n_y_***	**Number of weights**	**Inference time (*μs*)**	**Training (14 Subjects)**	**Validation (2 Subjects)**	**Test (1 Subject)**	**All**
1	20	3	5	1,046	46	91.1	85.5	80.6	89.9
1	20	6	6	1,466	45	90.9	85.5	80.0	89.6
1	25	5	5	1,556	42	91.5	83.6	73.9	89.5
1	20	6	3	1,106	40	92.3	76.1	75.5	89.4
1	25	6	5	1,681	41	91.3	81.1	78.2	89.3
1	25	4	7	1,731	40	90.2	83.4	83.7	89.0
1	20	4	4	1,026	41	90.0	84.6	80.5	88.8
1	30	2	3	1,056	41	90.0	80.2	79.1	88.2
1	20	6	5	1,346	51	86.7	85.5	82.4	86.3

**Figure 7:**
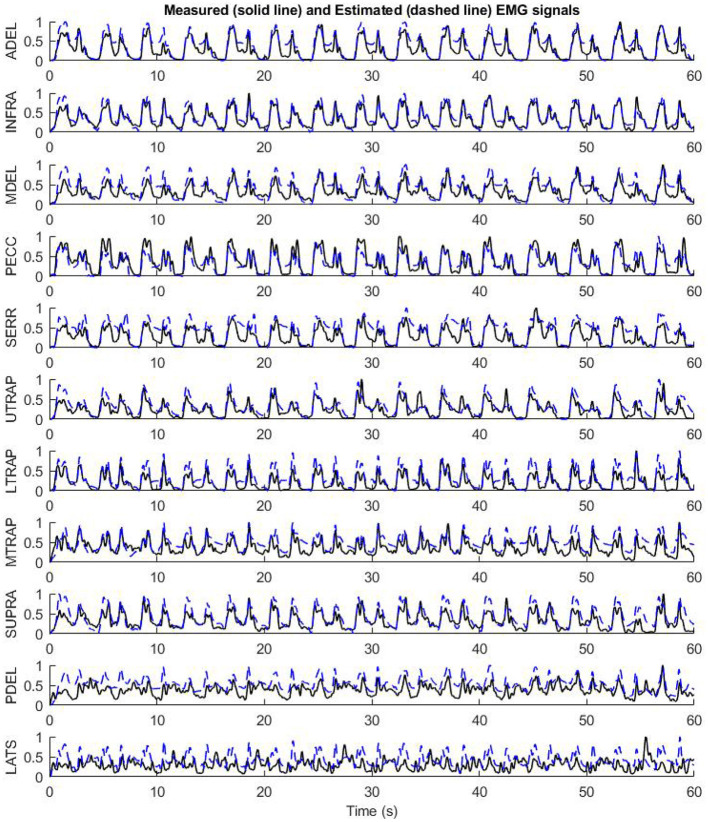
The performance of the preliminary configuration of InverseMuscleNET on test data to estimate EMG signals for 11 upper limb muscles.

An example of using the RNN model with the preliminary optimized configuration for mapping the biomechanical signals to EMG signals is shown in Figure 7. The validation subject has not participated in the training pool and represents a full prediction. As shown in Figure 7, the mapping has a good prediction of EMG signals in terms of timing and pattern. The subtle “off-pattern” observed may be due to insufficient data regarding external forces, like the target reaction load, which were not measured during the resting period of the experiment (Whittaker et al., 2018, 2019).

### 4.2. Evaluation of EMG Signal Estimation

**Figure 8:**
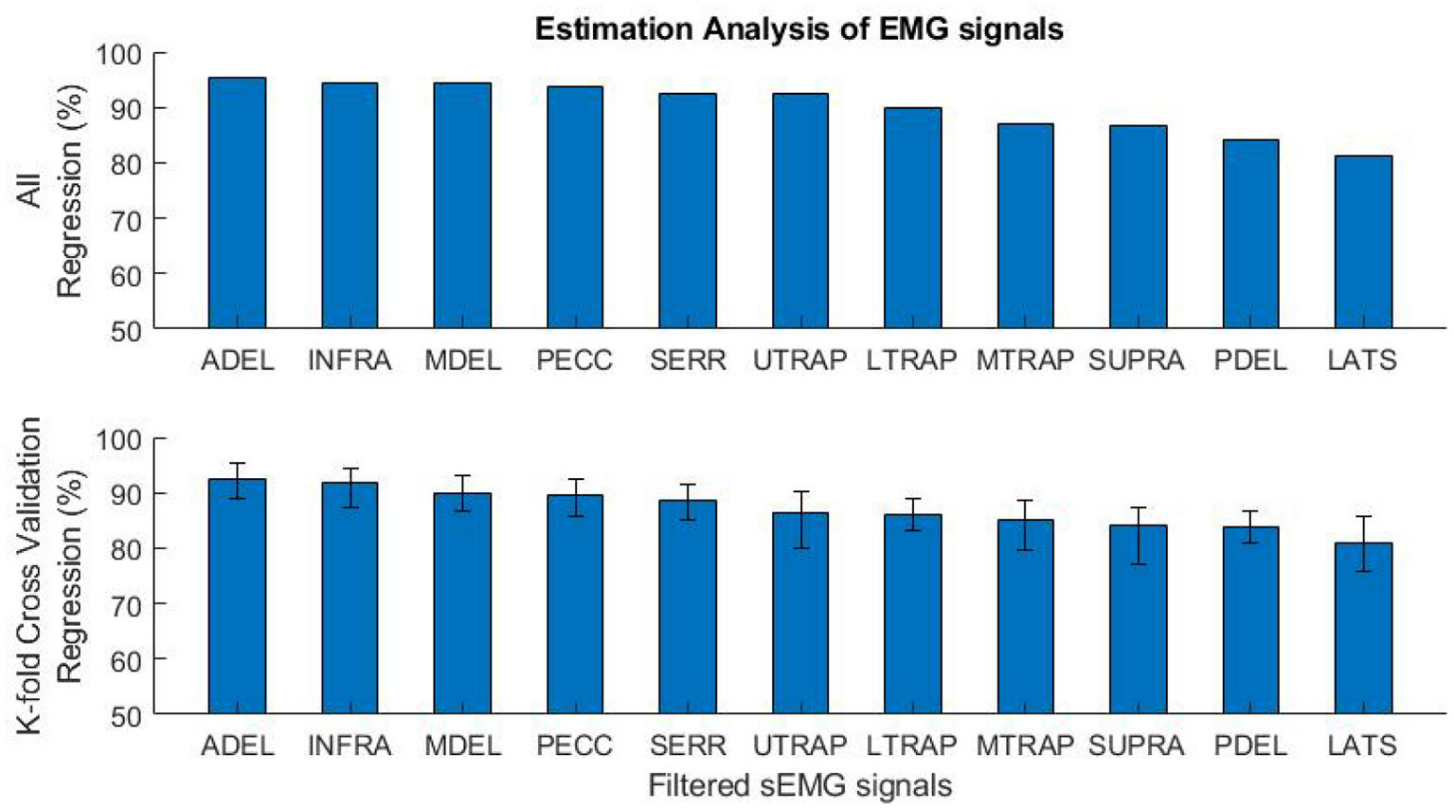
The mapping performance of 11 EMG signals for the general-model. All regression (training and validation) accuracies are shown on the top, and the K-fold cross-validation regression accuracies (average, maximum, and minimum) are shown on the bottom.

In Figure 8, the performance of biomechanical-EMG mapping is shown for each EMG signal. The performance of the general model has different results for each muscle. All regression (training-and-validation) and 17-fold cross-validation accuracies are shown in the top and the bottom of Figure 8, respectively. Although the regression accuracies have minor differences in both methods, the order of signal regression accuracy is the same. Signal predictions for each muscle (Figure 8) are shown from best to worst in the following order: Anterior Deltoid (ADEL), Infraspinatus (INFR), Middle Deltoid (MDEL), Pectoralis Major (PECC), Serratus Anterior (SERR), Upper Trapezius (UTRA), Lower Trapezius (LTRA), Middle Trapezius (MTRA), Supraspinatus (SUPR), Posterior Deltoid (PDEL), Latissimus Dorsi (LATS). This result does not mean that the dominant muscles, i.e., those primarily responsible for movement, are in the mentioned order. Some dominant muscles are located deeper under the skin, and recording with a surface electrode is subject to error and inefficiency. Rather, the order of dominant EMG contributions is based on the experimental data, motion, and measurements. For example, although the Latissimus Dorsi (LATS) EMG signal estimation performance was the lowest, the RNN model still showed good temporal predictions, i.e., following the shape of measured pattern and timing.

### 4.3. Input Biomechanical Signal Sensitivity Analysis

The results of the sensitivity analysis of the biomechanical signals are shown in Table 3. The analysis started from phase 1, which consists of all the biomechanical signals as the inputs. In the second phase, the analysis used four input signals as the inputs. The mapping performance minorly changed the evaluated regression from 90.0 to 87.9% when the joint acceleration signal was ignored. The joint acceleration, therefore, has a minor impact on the accuracy. For phase 3, four biomechanical signals (all except joint acceleration) were used for the mapping, and the joint velocity signals had the most negligible impact. These phases continued until all signals had been ignored for mapping. The order of most essential signals to least dominant ones was established from this protocol: joint angle, activation torque, joint torque, joint velocity, and joint acceleration. According to the mapping performance in Table 3, having all the biomechanical signals for mapping is helpful but not necessary.

**Table 3:** Sensitivity analysis of 5 input biomechanical signals.

		**Input signals**									
**Phase**	**Number of inputs**	**Joint angle**	**Joint velocity**	**Joint acceleration**	**Joint torque**	**Activation torque**	**ANN regression (%)**	**RNN regression (%)**	**order in phase**	**Signals order**	**Dominant Signals**
1	5	✓	✓	✓	✓	✓	83.9	91.0			
2	4	✓	✓	✓	✓		81.6	85.8	1		
	4	✓	✓	✓		✓	82.3	86.6	3		
	4	✓	✓		✓	✓	83.8	87.9	5	5	Joint acceleration
	4	✓		✓	✓	✓	82.6	87.0	4		
	4		✓	✓	✓	✓	82.1	86.0	2		
3	3	✓	✓		✓		79.9	83.2	2		
	3	✓	✓			✓	80.9	83.9	3		
	3	✓			✓	✓	81.0	84.4	4	4	Joint velocity
	3		✓		✓	✓	79.2	82.5	1		
4	2	✓			✓		75.8	82.0	2		
	2	✓				✓	76.0	82.9	3	3	Joint torque
	2				✓	✓	69.3	79.0	1		
5	1	✓					65.5	77.8	2	2	Activation torque
	1					✓	58.2	76.9	1	1	Joint angle

### 4.4. Final RNN Configuration Optimization

The final RNN configuration optimization utilized all five biomechanical signals and 6 of the 11 EMG signals. The configuration optimization was similar to section 3.4, but the 6 EMG signals consisted of Anterior Deltoid (ADEL), Infraspinatus (INFR), Middle Deltoid (MDEL), Pectoralis Major (PECC), Serratus Anterior (SERR), and Upper Trapezius (UTRA). These were selected according to the observations in section 4.2. Suppose the rest of the signals (those five with lower regression accuracy) are needed for specific clinical purposes/applications for different tasks. In that case, more data with different motions and tasks are required for training. The training properties were similar to the previous step. However, the maximum training time was set to 2 h, and the maximum epoch was set to 200 times. The training could be stopped after 100 epochs if the validation performance had not changed.

**Figure 9:**
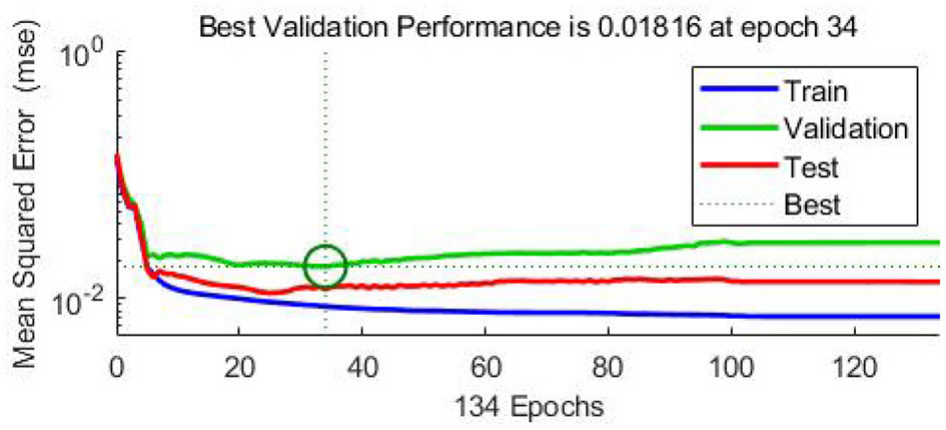
The RNN training (blue), validation (green), and test (red) performance (MSE) for 134 iterations. The exported model is based on epoch 34. The training stops after 100 iterations after the best validation performance.

**Figure 10:**
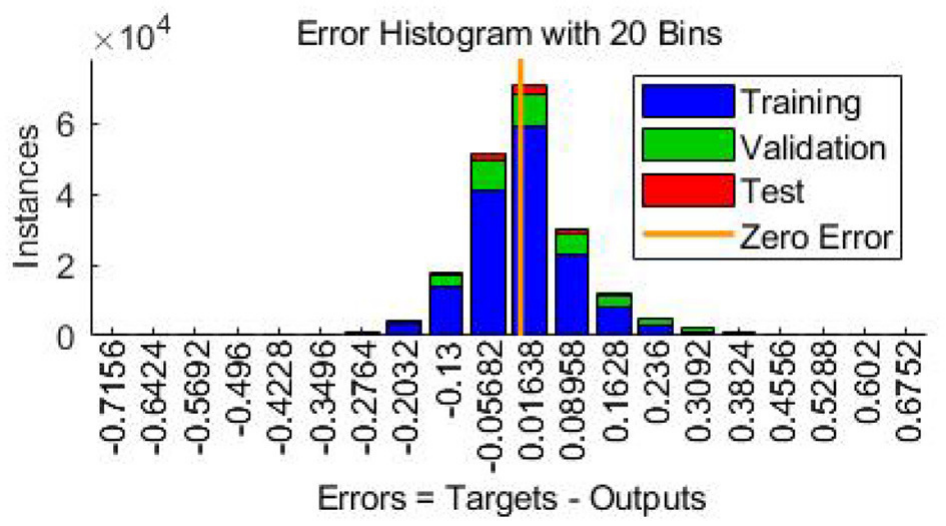
Error histogram indicating the difference between actual EMG signal and estimated output.

**Figure 11:**
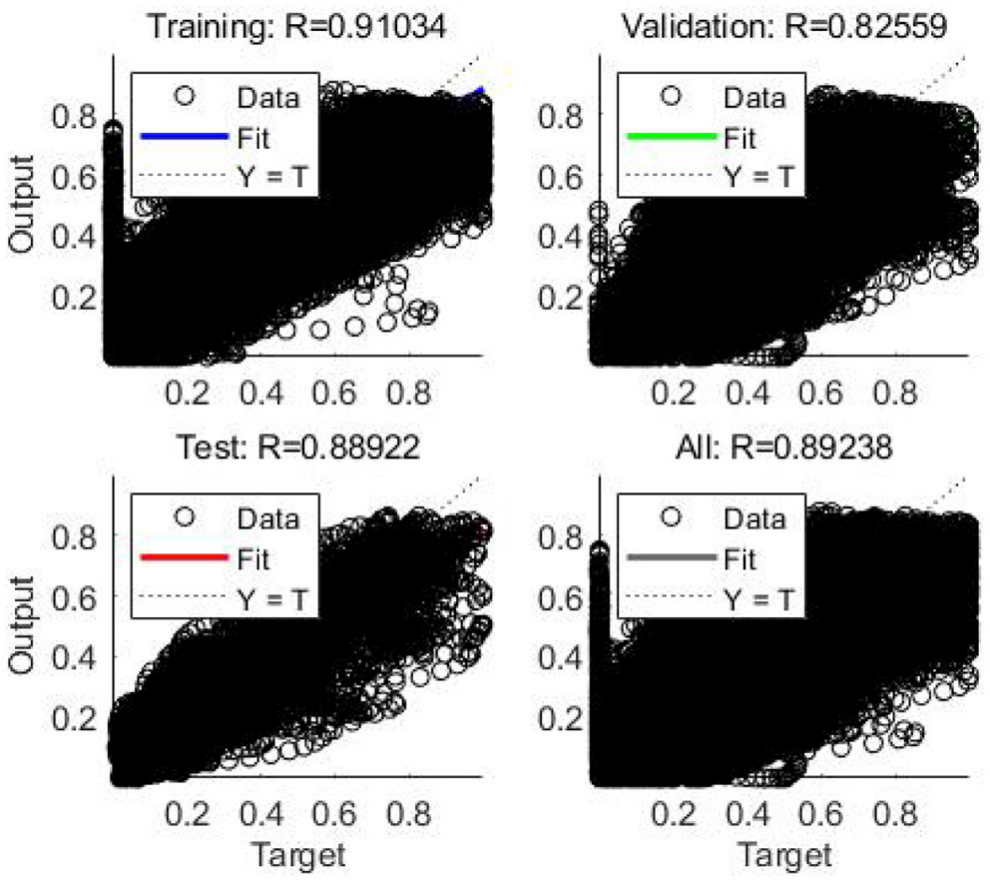
Regression or scatter plot of training, validation, test, and all data to RNN model.

In this case, the training was stopped after 134 iterations instead of 200 iterations since the best performance occurred at epoch 34 (Figure 9). The regression results for epoch 34 have been shown in Figure 10. The error histogram in Figure 11 indicates the difference between the measured EMG signal and the estimated output, which is primarily near zero and has a normal distribution. The regression accuracy for training, validation, test, and all sets are 91.0, 82.6, 88.9, and 89.2%, respectively.

From the optimization, the number of hidden layers (*n*_*l*_) was 1. The machine learning model consisted of two layers, one hidden layer, and one output layer. The number of nodes in the hidden layer (*n*_*n*_) was 20. The number of input signal former values (*n*_*u*_) was 3 and number of the output signal former values (*n*_*y*_) was 5. Since the sample rate of all input and output signals was 50 Hz, the input signals and the feedback signals should consist of 0.06 and 0.1 s of the vector input (containing current and previous states).

**Figure 12:**
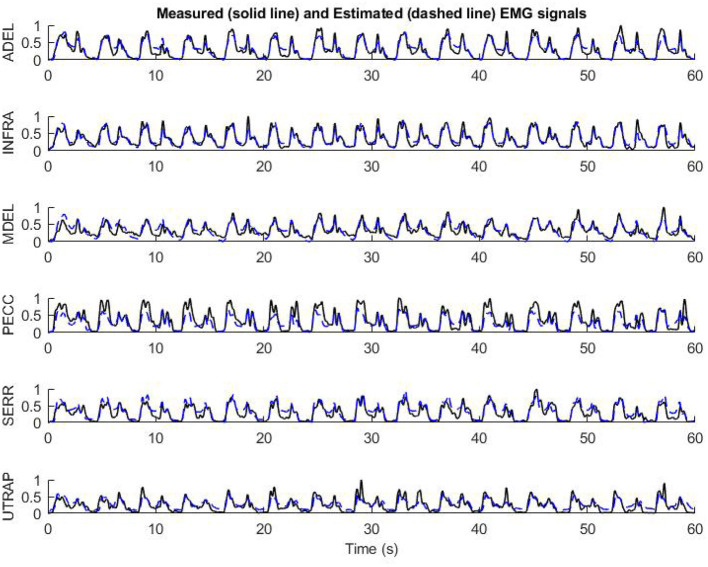
The performance of final optimized InverseMuscleNET configuration on test data for the estimation of EMG signals from 6 upper limb muscles.

For example, by feeding the biomechanical signals of the test subject to the model and having the initial value of EMG signal at 0 s, the estimation of 6 dominant EMG signals for 60 s has been presented in Figure 12. As can be seen, InverseMuscleNET with an optimized configuration could estimate the EMG signals quite well. The different amplitude of signals shows that the different subjects' muscles have varying strengths. The similar pattern of the estimated and measured signal indicates that muscle activation follows a similar pattern. Since the EMG signals have a stochastic behavior, time-varying nature, and are subject to measurement error, it is impossible to reach 100% accuracy for the estimation, and the achieved 90% regression is acceptable for biomechanical analysis.

The model's accuracy was slightly increased when using the elbow joint angle, velocity, and acceleration in addition to the previous shoulder joint angle, velocity, acceleration, torque, and activation torque. The training, validation, test, and all regression accuracy increased from 91.0, 82.6, 88.9, and 89.2% to 91.3, 83.3, 88.9, and 90.9%, respectively. The improvement is not much but can help the accuracy of the estimation model. We propose using the joint biomechanical variables relevant to the muscles that impact them.

## 5. Discussions

Optimal control and musculoskeletal modeling have been studied as potential tools for functional rehabilitation and sports evaluation. Despite significant progress, musculoskeletal modeling has encountered some significant problems. The muscle within a musculoskeletal system is complex and challenging to model, and the static optimization simulation requires a time-consuming iterative problem solution based on some assumptions (Williams and Constandinou, 2014; Bailly et al., 2021). Altogether, model development and simulation procedure are time-consuming, costly, and prone to error (Gonzalez-Vargas et al., 2015). Thus, it is challenging to use the model in clinical devices. Providing clinicians with a means of estimating EMG activity without an invasive electrode setup is the primary objective of this research.

This study intended to discover how to predict muscle activations accurately and efficiently using a machine learning model, specifically a recurrent neural network. Machine learning is an instrument or model for solving complicated mathematical problems without knowing the analytical relationship between inputs and outputs (Rane et al., 2019). However, there are a few disadvantages: it is challenging to determine the optimum network configuration and train the model; obtaining a rich set of input signals to solve the problem is also time-consuming. The main goal was to obtain the muscle activation solution with minor estimation errors and the fastest training convergence.

**Figure 13:**
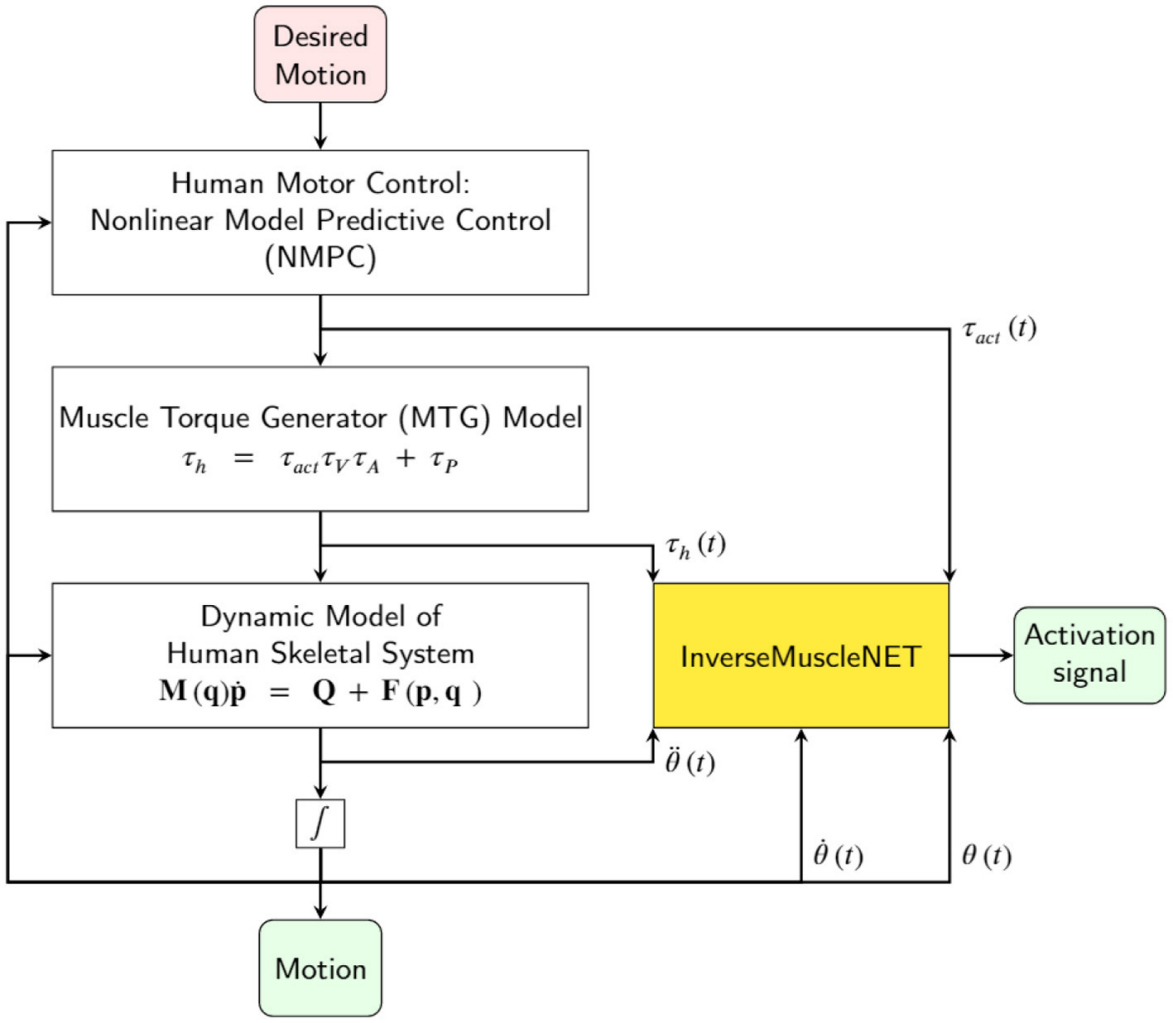
Schematic of using InverseMuscleNET within a forward dynamic simulation of the skeletal model for biomechanical analysis and sports engineering/optimization.

Recently, machine learning models have been developed to estimate skeletal muscle tensions without explicit modeling of the physical behaviors of muscles (Arjmand et al., 2013; Cecchini et al., 2014; Vilimek, 2014; Dao, 2019; Rane et al., 2019). However, an inverse muscle model has not been developed using a machine learning model. An optimization problem should be used to estimate the muscle activations since there is no inverse dynamic model of the muscles. A new modeling approach, which provides an inverse dynamic model of muscles, is proposed. This RNN model can offer the ability to compare the subject's EMG signals via estimated activation signals. Thus, clinicians can avoid EMG electrode setups in a laboratory and use the InverseMuscleNET. This inverse model completes the inverse dynamic simulation of the skeletal model for biomechanical analysis, post-rehabilitation analysis, and sports engineering/optimization. In addition, InverseMuscleNET can be used within a forward dynamic simulation of the skeletal model (Figure 13). In a forward dynamic simulation, the human motor control can be implemented by nonlinear model predictive control (Mehrabi et al., 2017). Other applications include modeling, functional electrical stimulation control, assessment and biofeedback, and biped robotic control (Gonzalez-Vargas et al., 2015).

The inverse muscle model was a NARX network, selected as an RNN learning model. The RNN includes a feedback connection to provide memory capacity for time-varying data. Successfully, Dao (2019) used an RNN for considering the dynamic temporal relationships of the muscle forces for the first time. We have shown that using an RNN could predict muscle activation by using joint kinematics and kinetics without additional resources.

Moreover, the RNN model was used to estimate muscle activation with fewer input signals in the sensitivity analysis section. These fewer input signals demonstrated the flexibility of the RNN model in the estimation and muscle model. This model could be trained based on the availability of input data, consisting of joint angle, velocity, acceleration, torque, and activation torque. It is noteworthy to mention that this RNN flexibility could not be done with static optimization approaches.

Developing a machine learning model has some challenging aspects, including (I) determining the optimal configuration and (II) requiring a rich and representative dataset for training. We have tested multiple topologies with the same training conditions to determine the model topology's optimal configuration. The number of hidden layers (1), the number of nodes in each layer (20), the number of input signal former values (3), and the number of the output signal former values (5) have been optimized to have the highest mapping accuracy. Although having a model with more parameters may have more accuracy, the model may have a delay and require more data for training. Having fewer parameters in the trained network allows the network to be saved and straightforwardly used in real-time.

Forming a rich database for model training was established on the data collection for a repetitive manual task from 17 healthy right-handed young individuals (Whittaker et al., 2018, 2019). Fourteen subjects' data was used for training, two subjects were used to validate the model, and one subject was used for testing the model. Since the validation dataset consisted of 2 subjects, we can be reasonably confident that the exported model was not overfitting the training dataset (Ying, 2019) and is general enough for assessing the upper limb motor task in question. The training dataset consisted of 14 subjects' datasets and is enough for training and finding the general-model since the full estimation has more than 90% regression accuracy. However, as the dataset increases, the final model becomes more general, reliable, and valuable. Since the data is recorded during object manipulation in the sagittal plane, it is not certain that the model can be used for other motions. The model is motion-based, and for a general-model, a large amount of data is required.

Another strength of this study relates to the rich number of involved muscles. Having access to 11 muscles provides the opportunity to evaluate the estimation possibility. The dominant and most accurate estimation occurred with the Anterior Deltoid (ADEL), Infraspinatus (INFR), Middle Deltoid (MDEL), Pectoralis Major (PECC), Serratus Anterior (SERR), and Upper Trapezius (UTRA) for the described task and motion.

Analysis of using five biomechanical signals for the estimation provides the possibility of sensitivity analysis. The order of most essential signals to least dominant ones was as follows: joint angle, activation torque, joint torque, joint velocity, and joint acceleration. This result demonstrated that the estimation of activation signals was relevant to joint angle, activation torque, and joint velocity. The results from the sensitivity analysis concur with previous classical muscle mathematical model studies. The muscle pennation angle and muscle-tendon length have been found to be sensitive to the joint angle and velocity (Winters, 1990). The maximum muscle force was relevant to the joint torque (Winters, 1990). Thus, the first three dominant signals correspond to the primary variables in the classical muscle mathematical model.

Since we found that the input signal and the feedback signal should consist of 0.06 and 0.1 s of the vector input (containing current and previous states) in the optimization of the RNN configuration process, the estimation of EMG signals relies on the history of the previous value of EMG signals and the biomechanical signals.

The estimated and the measured EMG signals in Figure 12 showed that the designed RNN model, in some cases, had slight differences. Occasionally, the measured EMG signals had small amplitude differences from the estimated muscle activation. However, the pattern of the estimated and measured signals is similar enough for the final applications, which are simulations or clinics. Sometimes, the measured EMG had high changes while the estimated variable followed a curved path, but the two signals had similar patterns. It is noteworthy to mention that every single computational technique for muscle variable computations has limitations in analytical expressions and suffers from unrealistic assumptions in the muscle models. The model parameters identified with measurements that are subject to error, require an iterative optimization loop, and results in estimated muscle activations that may not be correct (Norman-Gerum and McPhee, 2018).

The main limitation of the mentioned model for inverse muscle stimulation is the amount of training data. The machine learning model depends significantly on training data, like the human brain that tunes the neurons' weights and bias using motion and response. As far as we are concerned, there were 14 sets of input/target pairs from fourteen subjects, two to three subjects' data for validation, and one set for training. The data consisted of shoulder flexion/extension movement (the combination of external load, slow, and a fast motion), each of them in 12 trials of the pick-and-placing task. Having more data for more ranges of motion would facilitate a more general model.

## 6. Conclusion

A model estimating the shoulder muscle activation was developed using an optimized recurrent neural network. This estimation was based on biomechanical (kinematic and kinetic) input signals. This study suggests that the classical static optimization of the dynamic model could be replaced with an inverse muscle model using a machine learning model. This approach could increase potential decision support tools for functional rehabilitation with real-time estimation and muscle activation or EMG signal tracking.

## Data Availability Statement

The datasets presented in this article are not readily available due to ethical and privacy reasons. Requests to access the datasets should be directed to Clark R. Dickerson, clark.dickerson@uwaterloo.ca.

## Ethics Statement

The studies involving human participants were reviewed and approved by the University of Waterloo Office of Research Ethics. The patients/participants provided their written informed consent to participate in this study.

## Author Contributions

AN and JM contributed to the conception and design of the study. AN organized the interpretation of data, the model, and the code. AN, KI, SB, and JM performed the statistical analysis. AN, SB, and KI wrote the first draft of the manuscript and wrote sections of the manuscript. KI and JM performed revising it critically for important intellectual content. All authors contributed to manuscript revision, read, and approved the submitted version.

## Funding

This research is supported by funding from the Canada Research Chairs Program and the Natural Sciences and Engineering Research Council of Canada (NSERC).

## Conflict of Interest

The authors declare that the research was conducted in the absence of any commercial or financial relationships that could be construed as a potential conflict of interest.

## Publisher's Note

All claims expressed in this article are solely those of the authors and do not necessarily represent those of their affiliated organizations, or those of the publisher, the editors and the reviewers. Any product that may be evaluated in this article, or claim that may be made by its manufacturer, is not guaranteed or endorsed by the publisher.

## References

[B1] Anderson F. C. Pandy M. G. (2001). Static and dynamic optimization solutions for gait are practically equivalent. J. Biomech. 34, 153–161. 10.1016/s0021-9290(00)00155-x 11165278

[B2] Arjmand N. Ekrami O. Shirazi-Adl A. Plamondon A. Parnianpour M. (2013). Relative performances of artificial neural network and regression mapping tools in evaluation of spinal loads and muscle forces during static lifting. J. Biomech. 46, 1454–1462. 10.1016/j.jbiomech.2013.02.026 23541615

[B3] Avers D. Brown M. (2018). Daniels and Worthingham's Muscle Testing: Techniques of Manual Examination and Performance Testing, 10th Edn. (Madrid: Elsevier).

[B4] Bailly F. Ceglia A. Michaud B. Rouleau D. M. Begon M. (2021). Real-time and dynamically consistent estimation of muscle forces using a moving horizon EMG-marker tracking algorithm-Application to upper limb biomechanics. Front. Bioeng. Biotechnol. 9:642742. 10.3389/fbioe.2021.642742 33681174 PMC7928053

[B5] Bollen M. H. Gu I. Y. H. (2005). Signal Processing of Power Quality Disturbances. New York, NY: John Wiley & Sons. p.861

[B6] Cecchini G. Lozito G. M. Schmid M. Conforto S. Fulginei F. R. Bibbo D. (2014). Neural networks for muscle forces prediction in cycling. Algorithms 7, 621–634. 10.3390/a7040621

[B7] Crowninshield R. D. Brand R. A. (1981). A physiologically based criterion of muscle force prediction in locomotion. J. Biomech. 14, 793–801. 10.1016/0021-9290(81)90035-x 7334039

[B8] Dao T. T. (2019). From deep learning to transfer learning for the prediction of skeletal muscle forces. Med. Biol. Eng. Comput. 57, 1049–1058. 10.1007/s11517-018-1940-y 30552553

[B9] Desplenter T Trejos A. L.. (2018). Evaluating muscle activation models for elbow motion estimation. Sensors (Switzerland) 18, 1004. 10.3390/s18041004 29597281 PMC5948752

[B10] Dumas R. Chèze L. Verriest J. P. (2007). Adjustments to McConville and Young body segment inertial parameters. J. Biomech. 40, 543–553. 10.1016/j.jbiomech.2006.02.013 16616757

[B11] Ellis R. G. Rankin J. W. Hutchinson J. R. (2018). Limb kinematics, kinetics and muscle dynamics during the sit-to-stand transition in greyhounds. Front. Bioeng. Biotechnol. 6:162. 10.3389/fbioe.2018.00162 30505834 PMC6250835

[B12] Erdemir A. McLean S. Herzog W. van den Bogert A. J. (2007). Model-based estimation of muscle forces exerted during movements. Clin. Biomech. 22, 131–154. 10.1016/j.clinbiomech.2006.09.005 17070969

[B13] Ezati M. Ghannadi B. McPhee J. (2019). A review of simulation methods for human movement dynamics with emphasis on gait. Multibody Syst. Dyn. 47, 265–292. 10.1007/s11044-019-09685-1

[B14] Gonzalez-Vargas J. Sartori M. Dosen S. Torricelli D. Pons J. L. Farina D. (2015). A predictive model of muscle excitations based on muscle modularity for a large repertoire of human locomotion conditions. Front. Comput. Neurosci. 9:114. 10.3389/fncom.2015.00114 26441624 PMC4585276

[B15] Gurchiek R. D. Cheney N. McGinnis R. S. (2019). Estimating biomechanical time-series with wearable sensors: A systematic review of machine learning techniques. Sensors (Switzerland) 19, 5227. 10.3390/s19235227 31795151 PMC6928851

[B16] Hammer M. Günther M. Haeufle D. F. Schmitt S. (2019). Tailoring anatomical muscle paths: a sheath-like solution for muscle routing in musculoskeletal computer models. Math. Biosci. 311, 68–81. 10.1016/j.mbs.2019.02.004 30844381

[B17] Heidlauf T Rohrle O. (2014). A multiscale chemo-electro-mechanical skeletal muscle model to analyze muscle contraction and force generation for different muscle fiber arrangements. Front. Physiol. 5:498. 10.3389/fphys.2014.00498 25566094 PMC4274884

[B18] Heintz S. Gutierrez-Farewik E. M.. (2007). Static optimization of muscle forces during gait in comparison to EMG-to-force processing approach. Gait Posture 26, 279–288. 10.1016/j.gaitpost.2006.09.074 17071088

[B19] Heitmann S. Ferns N. Breakspear M. (2012). Muscle co-contraction modulates damping and joint stability in a three-link biomechanical limb. Front. Neurorobot. 5:5. 10.3389/fnbot.2011.00005 22275897 PMC3257849

[B20] Heller B. W. Veltink P. H. Rijkhoff N. J. Rutten W. L. Andrews B. J. (1993). Reconstructing muscle activation during normal walking: A comparison of symbolic and connectionist machine learning techniques. Biol. Cybern. 69, 327–335. 10.1007/BF00203129 8218535

[B21] Hirashima M. Oya T.. (2016). How does the brain solve muscle redundancy? Filling the gap between optimization and muscle synergy hypotheses. Neurosci. Res. 104, 80–87. 10.1016/j.neures.2015.12.008 26724372

[B22] Inkol K. A. Brown C. McNally W. Jansen C. McPhee J. (2020). Muscle torque generators in multibody dynamic simulations of optimal sports performance. Multibody Syst. Dyn. 50, 435–452. 10.1007/s11044-020-09747-9

[B23] Jonic S. Popovic D. (1997). Machine learning for prediction of muscle activations for a rule-based controller, in Annual International Conference of the IEEE Engineering in Medicine and Biology-Proceedings, Vol. 4 (Chicago, IL: IEEE), 1781–1784.

[B24] Laschowski B. Mehrabi N. McPhee J. (2018). Optimization-based motor control of a paralympic wheelchair athlete. Sports Eng. 21, 207–215.

[B25] Lee J. H. Shin J. Realff M. J. (2018). Machine learning: overview of the recent progresses and implications for the process systems engineering field. Comput. Chem. Eng. 114, 111–121. 10.1016/j.compchemeng.2017.10.008

[B26] Manal K. Buchanan T. S.. (2003). A one-parameter neural activation to muscle activation model: estimating isometric joint moments from electromyograms. J. Biomech. 36, 1197–1202. 10.1016/s0021-9290(03)00152-0 12831746

[B27] McNally W. McPhee J. (2018). Dynamic optimization of the golf swing using a six degree-of-freedom biomechanical model. Proceedings 2, 243. 10.3390/proceedings2060243

[B28] Mehrabi N. McPhee J. (2019). Model-based control of biomechatronic systems, in Handbook of Biomechatronics (San Diego, CA: Academic Press), 95–126.

[B29] Mehrabi N. Razavian R. S. Ghannadi B. McPhee J. (2017). Predictive simulation of reaching moving targets using nonlinear model predictive control. Front. Comput. Neurosci. 10:143. 10.3389/fncom.2016.00143 28133449 PMC5233688

[B30] Michaud F. Shourijeh M. S. Fregly B. J. Cuadrado J. (2020). Do muscle synergies improve optimization prediction of muscle activations during gait? Front. Comput. Neurosci. 14:54. 10.3389/fncom.2020.00054 32754024 PMC7366793

[B31] Millard M. Emonds A. L. Harant M. Mombaur K. (2019). A reduced muscle model and planar musculoskeletal model fit for the simulation of whole-body movements. J. Biomech. 89, 11–20. 10.1016/j.jbiomech.2019.04.004 31000347

[B32] Miller R. H. Brandon S. C. Deluzio K. J. (2013). Predicting sagittal plane biomechanics that minimize the axial knee joint contact force during walking. J. Biomech. Eng. 135, 011007. 10.1115/1.4023151 23363218

[B33] Moissenet F. Bélaise C. Piche E. Michaud B. Begon M. (2019). An optimization method tracking EMG, ground reactions forces, and marker trajectories for musculo-tendon forces estimation in equinus gait. Front. Neurorobot. 13:48. 10.3389/fnbot.2019.00048 31379547 PMC6646662

[B34] Nasr A. Bell S. He J. Whittaker R. L. Jiang N. Dickerson C. R.. (2021a). MuscleNET: Mapping electromyography to kinematic and dynamic biomechanical variables. J. Neural Eng. 18, 0460d3. 10.1088/1741-2552/ac1adc 34352741

[B35] Nasr A. Ferguson S. McPhee J. (2021b). Model-based design and optimization of passive shoulder exoskeletons, in Proceedings of the ASME 2021 Virtual International Design Engineering Technical Conferences & Computers and Information in Engineering Conference, DETC2021–69437, Online, Virtual. ASME.

[B36] Nasr A. He J. Jiang N. McPhee J. (2020). Activation torque estimation of muscles by forward neural networks (Forward-MuscleNET) for sEMG-based control of assistive robots, in Proceedings of the 7th International Conference of Control, Dynamic Systems, and Robotics (CDSR'20) 146, Virtual Conference.

[B37] Nasr A. He J. Jiang N. McPhee J. (2021c). Muscle modelling using machine learning and optimal filtering of sEMG signals, in Proceedings of the 45th Meeting of the American Society of Biomechanics (ASB2021), 89–91.

[B38] Norman-Gerum V. McPhee J. (2018). Constrained dynamic optimization of sit-to-stand motion driven by Bézier curves. J. Biomech. Eng. 140, 121011. 10.1115/1.4041527 30458529

[B39] Prentice S. D. Patla A. E. Stacey D. A. (2001). Artificial neural network model for the generation of muscle activation patterns for human locomotion. J. Electromyogr. Kinesiol. 11, 19–30. 10.1016/s1050-6411(00)00038-9 11166605

[B40] Rane L. Ding Z. McGregor A. H. Bull A. M. (2019). Deep learning for musculoskeletal force prediction. Ann. Biomed. Eng. 47, 778–789. 10.1007/s10439-018-02190-0 30599054 PMC6445355

[B41] Reaz M. B. I. Hussain S. Mohd-Yasin F. (2006). Techniques of EMG signal analysis: detection, processing, classification and applications. Biol. Procedures Online 8, 11–35. 10.1251/bpo115 16799694 PMC1455479

[B42] Sekiya M. Sakaino S. Toshiaki T. (2019). Linear logistic regression for estimation of lower limb muscle activations. IEEE Trans. Neural Syst. Rehabil. Eng. 27, 523–532. 10.1109/TNSRE.2019.2898207 30763243

[B43] Serrancolí G. Kinney A. L. Fregly B. J. (2020). Influence of musculoskeletal model parameter values on prediction of accurate knee contact forces during walking. Med. Eng. Phys. 85, 35–47. 10.1016/j.medengphy.2020.09.004 33081962

[B44] Shourijeh M. S. McPhee J. (2014). Forward dynamic optimization of human gait simulations: a global parameterization approach. J. Comput. Nonlin. Dyn. 9, 031018. 10.1115/1.4026266

[B45] Shourijeh M. S. Mehrabi N. McPhee J. (2017). Forward static optimization in dynamic simulation of human musculoskeletal systems: a proof-of-concept study. J. Comput. Nonlin. Dyn. 12, 051005. 10.1115/1.4036195

[B46] Siebert T. Rode C. Herzog W. Till O. Blickhan R. (2008). Nonlinearities make a difference: comparison of two common hill-type models with real muscle. Biol. Cybern. 98, 133–143. 10.1007/s00422-007-0197-6 18049823

[B47] Sohane A. Agarwal R. (2020). Knee muscle force estimating model using machine learning approach. The Comput. J. bxaa160.

[B48] Tibold R. Fuglevand A. J. (2015). Prediction of muscle activity during loaded movements of the upper limb. J. NeuroEng. Rehabil. 12, 1–12. 10.1186/1743-0003-12-6 25592397 PMC4326445

[B49] Valente G. Pitto L. Testi D. Seth A. Delp S. L. Stagni R.. (2014). Are subject-specific musculoskeletal models robust to the uncertainties in parameter identification? PLoS ONE 9:e112625. 10.1371/journal.pone.0112625 25390896 PMC4229232

[B50] Vilimek M. (2014). An artificial neural network approach and sensitivity analysis in predicting skeletal muscle forces. Acta Bioeng. Biomech. 16, 119–127. 10.5277/abb140314 25307446

[B51] Whittaker R. L. La Delfa N. J. Dickerson C. R. (2019). Algorithmically detectable directional changes in upper extremity motion indicate substantial myoelectric shoulder muscle fatigue during a repetitive manual task. Ergonomics 62, 431–443. 10.1080/00140139.2018.1536808 30321104

[B52] Whittaker R. L. Park W. Dickerson C. R. (2018). Application of a symbolic motion structure representation algorithm to identify upper extremity kinematic changes during a repetitive task. J. Biomech. 72, 235–240. 10.1016/j.jbiomech.2018.02.027 29523349

[B53] Williams I. Constandinou T. G.. (2014). Computationally efficient modeling of proprioceptive signals in the upper limb for prostheses: a simulation study. Front. Neurosci. 8:181. 10.3389/fnins.2014.00181 25009463 PMC4069835

[B54] Winter D. A. (2009). Biomechanics and Motor Control of Human Movement, 4th Edn. Hoboken, NJ: John Wiley & Sons.

[B55] Winters J. M. (1990). Hill-based muscle models: a systems engineering perspective, in Multiple Muscle Systems (New York, NY: Springer), 69–93.

[B56] Ying X. (2019). An overview of overfitting and its solutions. J. Phys. Conf. Series 1168, 022022. 10.1088/1742-6596/1168/2/022022

